# A Portable Sensor System with Ultramicro Electrode Chip for the Detection of Heavy-Metal Ions in Water

**DOI:** 10.3390/mi12121468

**Published:** 2021-11-28

**Authors:** Yuekun Wang, Yuhao Xu, Jinhua Jiang, Yang Li, Jianhua Tong, Chao Bian

**Affiliations:** 1 State Key Laboratory of Transducer Technology, Aerospace Information Research Institute, Chinese Academy of Sciences, Beijing 100190, China; wangyuekun19@mails.ucas.ac.cn (Y.W.); xuyuhao15@mails.ucas.ac.cn (Y.X.); jiangjinhua19@mails.ucas.ac.cn (J.J.); jhtong@mail.ie.ac.cn (J.T.); 2 School of Electronic, Electrical and Communication Engineering, University of Chinese Academy of Sciences, Beijing 100049, China

**Keywords:** MEMS, ultramicro interdigital electrode chip, heavy-metal ion, portable system, water quality detection

## Abstract

In this study, an ultramicro interdigital electrode array chip (UIEA) was designed and fabricated by using Micro-Electro-Mechanical systems (MEMS) technology, and a portable detection system, using the chip for determination of heavy-metal ions in water, was developed. The working electrode of the UIEA was modified with gold nanoparticles by electrodeposition. The detection sensitivity of the UIEA chip for copper ions was 0.0138 μA·L·μg^−1^, with the linear range of 0–400 μg/L and the detection limit of 18.89 μg/L (3σ), which was better than that of the compared columnar glassy carbon electrode. The results of the interference experiment verified that the UIEA chip has a certain anti-interference ability against common heavy-metal ions in water, such as Pb^2+^, Zn^2+^, and Mg^2+^ ions. The standard addition method was used to investigate the performance of the developed s ystem for copper ion determination in real water. The recovery range from 87.5% to 94.7% was achieved.

## 1. Introduction

Heavy-metal ions are reported to be low-density, but highly toxic, chemicals. Heavy metals are elements with an atomic density greater than 4.5 g/cm^3^, which are some of the most persistent pollutants in waste water. Excessive heavy-metal ions bring many negative impacts on the ecosystem, such as death of aquatic organisms, proliferation of algae, and destruction of animal and plant habitats [1].

Some kinds of heavy-metal ions, such as iron, copper, zinc, cobalt, and manganese, etc., are trace heavy-metal elements needed by organisms, but they will lead to toxic effects when their concentrations are too high in vivo. Other heavy-metal ions, such as cadmium, lead, and mercury, etc., are characterized as being highly toxic, even if they are ingested in small amounts. According to the World Health Organization’s (WHO) drinking water quality standard, the concentration of copper ions in drinking water should not exceed 2 mg/L; the concentration standards of various heavy-metal ions in water quality are shown in Table 1.

Drinking water with an excessive concentration of heavy-metal ions will trigger great harm to the human body. Excessive accumulation of heavy metals in the human body will reduce the energy level, and damage the functions of the brain, lungs, kidney, liver, blood components, and other important organs. As a result, the body, especially muscles and nerves, gradually develops degenerative diseases, such as multiple sclerosis, Parkinson’s disease, Alzheimer’s disease, and muscular dystrophy [2,3,4,5,6].

In recent years, more and more accidents have been reported that were caused by excessive heavy-metal ions in China. Among the eighteen major environmental incidents in China from 2012 to 2017, there were six heavy-metal pollution incidents, accounting for thirty-three percent [7]. This situation shows the seriousness of heavy-metal pollution and the necessity for detection of them. Most heavy metals exist in the form of inorganic ions in water, which are colorless and tasteless. They are generally difficult to detect directly, and high-precision instruments are necessary to detect them [8].

The traditional detection methods for heavy-metal ions mainly include atomic absorption spectrometry (AAS), inductively coupled plasma mass spectrometer (ICP-MS), surface-enhanced Raman scattering (SERS), ultraviolet and visible spectrophotometry (UV-Vis), and electrochemistry [9]. Although the former methods show high accuracy, they have some disadvantages in applications, such as the high cost of detection equipment, and the single species of detected ions. In addition, because the testing equipment is bulky, they are not suitable for on-site testing [10]. Electrochemistry, which has the advantages of low detection cost, high sensitivity, convenience, portability, and strong on-site detection capability [11,12,13], is widely used in the field of detecting heavy metals in the aquatic environment. The electrochemical method commonly used for heavy-metal detection is square-wave pulse voltammetry (SWV). Its waveform is composed of step scanning and a symmetrical bipolar pulse superimposed on each step, with one positive pulse and one reverse pulse. This method has the advantages of a wide potential window, small background interference, and fast scanning speed, so it is considered to be an efficient detection method [14]. Nanomaterials are often used to modify the surface of electrochemical sensors, and to enhance the performance of electrodes because of their high conductivity and stability [15]. For example, single-walled carbon nanotubes and gold nanoparticles were used to modify the electrode surface of a disposable sensor to optimize the sensor performance for lead (Pb^2+^) determination [16]. Arcos-Martínez et al. used a nano-platinum-modified, carbon-based, and screen-printed electrochemical sensor to detect arsenic (As^3+^) [17].

There is also some progress in the research on portable heavy-metal-ion detection systems using the electrochemical method. Orawon Chailapakul et al. used paper-based sensors combined with a commercial portable electrochemical reader (Metrohm DropSens, Spain) to detect tin and lead simultaneously [18]. Elena Bernalte et al. implemented the detection of copper in the Amazon River with a single screen-printed electrode probe. The instrument was a commercial, hand-held, and battery-powered PalmSens4 potentiostat, which could record the data and transfer them to a mobile device via a wireless connection. The detection limit of that electrode for copper ions was 1.5 μg/L, with the linear range of 5~300 μg/L [8]. Wang et al. designed a miniaturized electrochemical system and a screen-printed carbon electrode with a gold-nanoparticle modification for the determination of chromium (VI). The electrochemical system was composed of an analyzer, a detection module, and a laptop or smartphone. The results of the detection showed a sensitivity of 1.1 nA·L·μg^−1^, and a limit of detection of 5.4 μg/L for chromium ions [19]. Lin et al. developed a smartphone-based water-quality monitoring system with a whole-copper electrochemical sensor chip for the quantification of lead ions. A hand-held detector was used to perform the electrochemical measurements, record the measured data, and send them to the smartphone. The system could detect lead ions in water as low as 9.3 μg/L [20].

In order to design a detection system to realize rapid detection of heavy metals in water, this study proposed a portable heavy-metal-ion sensing and detection system based on anodic stripping voltammetry, which is more miniaturized in size, and lower in energy consumption. The developed system was used to detect copper ions in water, and the experimental results revealed that the system had the advantages of miniaturization and portability, and was suitable for on-site rapid detection of heavy-metal ions.

## 2. Materials and Methods

### 2.1. Instruments and Reagents

A Gamry Reference 600 electrochemical workstation (Gamry, Warminster, PA, USA), electronic balance (Sartorius, Göttingen, Germany), ultrapure water machine (Beijing Yingan Meicheng Scientific Instrument Co., Ltd., Beijing, China), and silver/silver chloride electrode (Shanghai Chenhua Instrument Co., Ltd., Shanghai, China) were employed.

Potassium chloride (KCl), potassium ferricyanide (K_3_[Fe(CN)_6_]), chloroauric acid (HAuCl_4_), and anhydrous sodium acetate (CH_3_COONa) were purchased from Sinopharm Chemical Reagent Co., Ltd. (Shanghai, China); potassium ferrocyanide (K_4_[Fe(CN)_6_]) was purchased from Xilong Chemical Co., Ltd. (Shantou, China); acetic acid (CH_3_COOH) was purchased from Beijing Chemical Plant Co., Ltd. (Beijing, China); and copper standard solution was purchased from the National Analysis and Testing Center for Nonferrous Metals and Electronic Materials. All experimental reagents were analytically pure, and the experimental water was deionized water made from ultrapure water. Unless otherwise specified, the experimental temperature conditions were room temperature (25 °C).

### 2.2. Ultramicro Interdigital Electrode Chip

Ultramicro interdigital electrodes are small, and generally refer to electrodes with micron or even nanometer wire diameters. Multiple microelectrodes are arranged and connected together in a certain way to form a microelectrode array, which can show better electrochemical characteristics.

**Figure 1:**
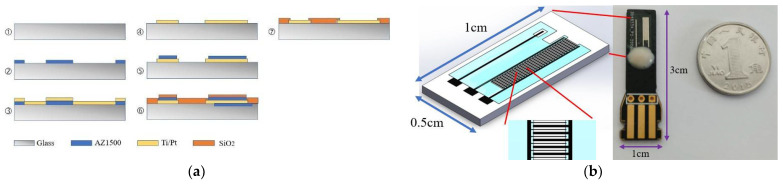
Ultramicro interdigital array electrode chip: (**a**) fabrication process; (**b**) schematic and picture of the electrode chip.

The fabrication process of the ultramicro electrode array chip is shown in Figure 1a. Micro-Electro-Mechanical systems (MEMS) technology was used to prepare the sensing electrode chip with an ultramicro interdigital array structure that integrated the working electrode and the counter electrode. A glass wafer with good insulation characteristics was used as the substrate. Firstly, the positive photoresist AZ1500 was coated on the glass substrate, and formed the pattern of the ultramicro array electrode by photolithography. Next, titanium (Ti) with a thickness of 20 nm was sputtered as the adhesion layer, and then platinum (Pt) with a thickness of 200 nm was sputtered as the electrode layer, and the pattern transfer was completed by the lift-off process. After that, silicon oxide as a waterproof insulation layer with the thickness of 1 μm was prepared by the method of plasma enhanced chemical vapor deposition (PECVD). Then, patterning of the insulation layer to define the effective area of the electrode was implemented by the lithographic and lift-off processes. Finally, after dicing and packaging, the ultramicro electrode array chip was fabricated and ready for use.

For easy use, the ultramicro electrode array chip was bonded and packaged on a PCB board with the thickness of 0.6 mm, the width of 1 cm, and the length of 3 cm. A picture of the electrode chip and the schematic of the ultramicro interdigital electrode array chip are shown in Figure 1b. The ultramicro interdigital electrode array chip, with the width of 0.5 cm and the length of 1 cm, has 30 units of working electrode and counter electrode. The working electrode is composed of a rectangular array, and each rectangle is 15 μm in width and 1000 μm in length. The counter electrode has a similar shape, but the area of the rectangular unit is larger, with the width of 60 μm and length of 1000 μm. The finger spacing between the working electrode unit and the counter electrode unit is 60 μm. The total sensing area of the working electrode is 0.45 mm^2^. A KCl-saturated Ag/AgCl electrode (CHI111, CH Instruments, Shanghai, China) was used as the reference electrode (RE) to form a three-electrode system with the fabricated electrode chip.

A columnar glassy carbon electrode and a columnar platinum electrode were used as the working electrode and the counter electrode in comparison experiments. In the following experiments, gold nanoparticles were electrodeposited on the working electrode of the ultramicro interdigital electrode chip and on the columnar glassy carbon electrode by the constant potential method, which were used as the sensing material for copper ions determination. The deposition potential was −0.2 V and the deposition time was 300 s. The concentrations of copper ions were detected by both the ultramicro interdigital electrode chip and the commonly used columnar electrode.

### 2.3. System Hardware Design

**Figure 2:**
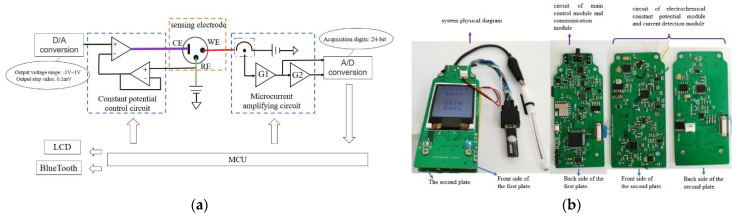
Portable heavy-metal-ion sensor detection system: (**a**) system block diagram; (**b**) picture of the detection circuit unit.

The portable sensing and detection system mainly consists of two parts: a heavy-metal-ion sensing electrode and a detection circuit unit; the structural framework is shown in Figure 2a. The system has a length of 9 cm, a width of 3.5 cm, and a thickness of 1.5 cm. The detection circuit unit is composed of a main control module, an electrochemical constant potential module, a current detection module, and a communication module. The picture of the detection circuit is shown in Figure 2b.

The main control module uses a 32-bit high-performance STM32F405RGT6 chip as the microcontroller. Its working voltage range is from 1.8 V to 3.6 V. Its chip package size is small, which can reduce the power consumption of the equipment, and is suitable for portable equipment. This module also includes a clock system, a program downloading and debugging circuit, and a power-on reset circuit.

In the three-electrode system, it is required that the potential between the reference electrode and the working electrode is constant, and that no current flows through the reference electrode. An electrochemical potentiostat is used to maintain the constant voltage between the reference electrode and the working electrode, and to control the required voltage mode. The core of the circuit is a comparison amplifier, which is composed of a deep negative feedback differential amplifier, including a digital-to-analog converter (DAC) and three operational amplifiers. The digital-to-analog converter uses a DAC8552 chip to generate a bipolar square wave waveform and variable pulse voltage.

The current detection module detects the response current signal generated by the sensing electrode, and adopts a multi-stage series of amplification to improve the precision of current detection. Due to the large amplitude span of the response current, in order to ensure the accuracy of the measurement, it is necessary to design a detection circuit with a variable range or multi-stage amplification. Compared with a single-channel programmable-gain amplification circuit, this kind of design can realize a different gain amplification without using the channel selector to switch the feedback resistor, and the single-stage amplification gain of the operational amplifier can be controlled below 100 times, which has better amplification characteristics. The program-controlled, multi-channel acquisition unit uses an AD7124 chip to realize the acquisition and analog-to-digital conversion of the output voltage of the multi-stage series gain link.

The communication module mainly transmits data to the upper computer or mobile intelligent terminal in real time through Bluetooth and a serial port. After the current collection and data analysis, the corresponding concentration value of heavy-metal ions can be obtained, and the testing process can be displayed on the LCD screen.

### 2.4. System Software Design

**Figure 3:**
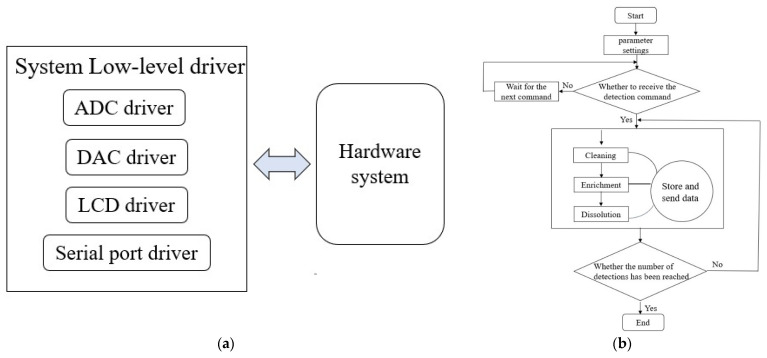
The program of the embedded software system: (**a**) design block diagram; (**b**) flowchart.

The program design of the underlying driver for the hardware detection system was developed in a Keil MDK5 integrated development environment, which is specially designed for microcontroller applications. According to the modular design of the hardware system, the program of the embedded software system mainly consists of an ADC (analog-to-digital conversion) drive, a DAC (digital-to-analog conversion) drive, a serial port drive, and an LCD display drive. The program design block diagram and flow chart are shown in Figure 3.

The ADC driver calculates the voltage value of the analog signal by the reverse calculation of the digital signal, and uses an SPI communication protocol to read and write data. The program includes the AD7124 chip initialization, single-conversion data reading, sampling data compensation, and sampling data uploading. The DAC driver includes the DAC8552 chip initialization program, digital output program, and analog output program. Because the communication mode between the Bluetooth chip and the hardware system is serial port communication, a serial port driver is used to send and receive instructions and the data of the host computer, mainly including the serial port initialization function and the serial port interrupt function, and data transmission adopts a unified format. The LCD driver mainly consists of the initialization function and the display character function of the LCD display chip.

## 3. Results and Discussion

### 3.1. Gold Nanoparticle Modification

**Figure 4:**
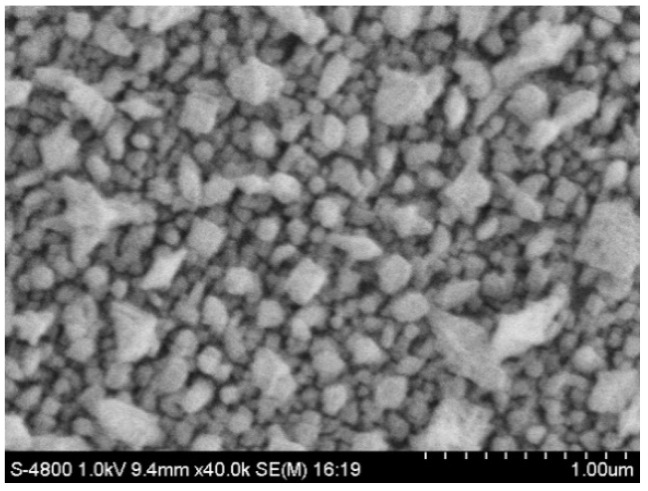
Surface morphology of the ultramicro electrode modified with gold nanoparticles.

The SEM image of the surface of the ultramicro electrode modified with gold nanoparticles is shown in Figure 4. It was found that the morphology of the fabricated gold nanoparticles was dense.

**Figure 5:**
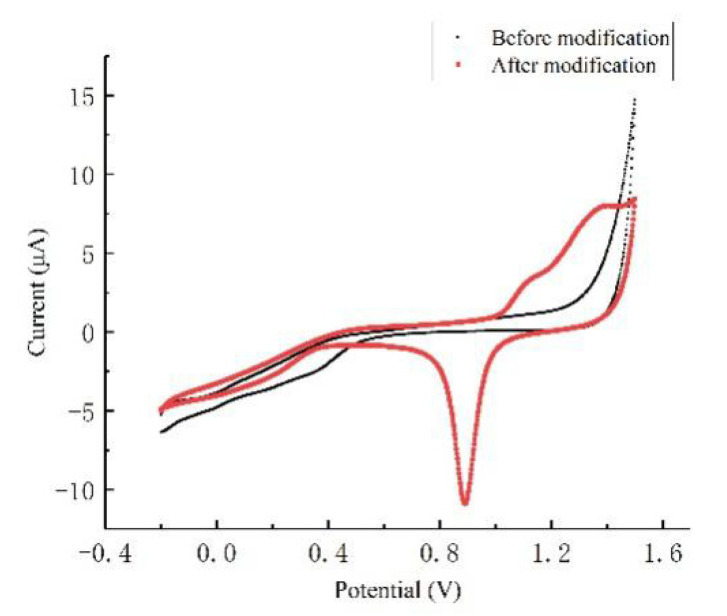
CV characteristic curve of the ultramicro electrode in H_2_SO_4_ before and after modification.

The cyclic voltammogram of the ultramicro interdigital array electrode in 0.05 M sulfuric acid (H_2_SO_4_) solution before and after modification was shown in Figure 5. It was found that, compared with the CV scanning curve before modification of nano-gold, the CV scanning curve after modification had a reduction current peak of gold between 0.85 V and 0.9 V, which verified that gold nanoparticles were modified onto the electrode surface, and showed the characteristic curve of cyclic voltammetry scanning of gold in sulfuric acid [21].

**Figure 6:**
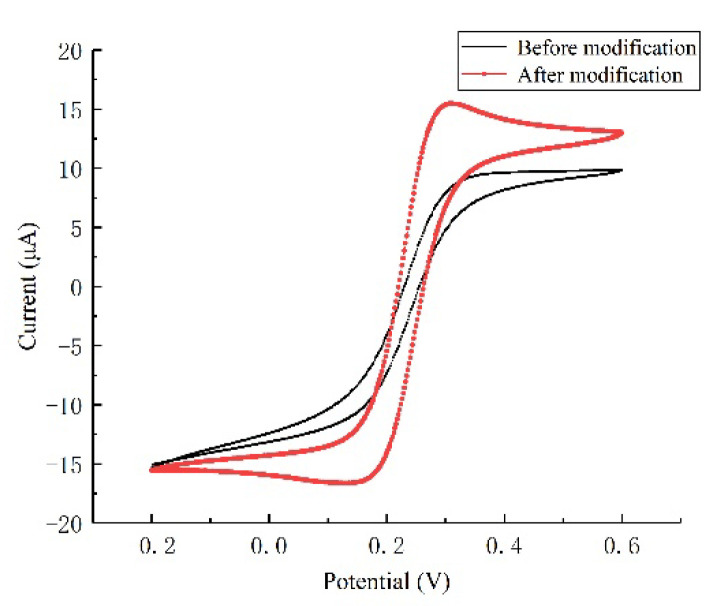
CV characteristic curve of the ultramicro electrode in K_3_[Fe(CN)_6_], before and after modification.

Ultramicro interdigital array electrodes, with and without the modification of gold nanoparticles, were both immersed in a 5 mM potassium ferricyanide (K_3_[Fe(CN)_6_]) solution for cyclic voltammetry scanning. The scanning potential range was from −0.2 V to 0.6 V, and the scan rate was 50 mV/s. The cyclic voltammetry scan curves before and after the modification process are shown in Figure 6. The ultramicro interdigital array electrode exhibited the typical ‘S’ characteristic curve. The oxidation-reduction current remained basically stable after reaching the oxidation and reduction potential, which reflected the hemispherical diffusion and the rapid mass transfer characteristics of the ultramicroelectrode. Better electrochemical performance was exhibited by the ultramicro interdigital array electrode modified with gold nanoparticles. The redox peak current increased after the modification process. 

### 3.2. Optimization of Detection Parameters

**Figure 7:**
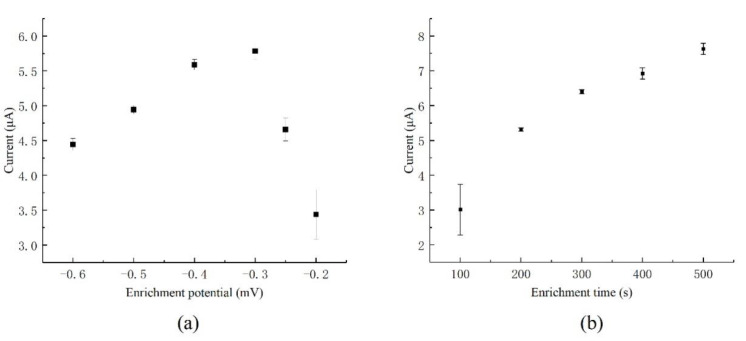
Optimization of detection parameters for the ultramicro interdigital electrode: (**a**) optimization of enrichment potential; (**b**) optimization of enrichment time. (The detection results are the average of three times.).

In the enrichment stage, a constant negative voltage is applied to the working electrode and the heavy-metal ions could be reduced and deposited on the surface of the working electrode. Therefore, it is necessary to optimize the parameters related to the enrichment process for the detection. In the sample with a copper ion concentration of 400 μg/L, the enrichment voltage and enrichment times were both optimized, and the results are shown in Figure 7.

As shown in Figure 7a, with enrichment time of 300 s, with the negative shift of the enrichment potential from −0.2 V to −0.3 V, the current response tended to increase, while the error bar in these tests tended to decrease. It showed that as the potential shifted negatively, the amount of the deposited copper ions on the electrode surface increased. In addition, the deposition of copper ions became more and more stable. With the further negative shift of the enrichment potential, the hydrogen evolution reaction arose on the surface of the electrode, which reduced the enrichment efficiency, and the dissolution current gradually decreased. Considering the intensity of the stripping current and the consistency of the enrichment dissolution, −0.5 V was selected as the enrichment potential.

As shown in Figure 7b, with the enrichment time increasing from 100 s to 500 s, the stripping currents increased gradually, which means the increasing of deposited gold nanoparticles. With an enrichment time of 100 s, poor repeatability was observed, which may imply that the deposited gold film was unstable. When the enrichment time was over 400 s, the repeatability became poor with a high standard deviation. It may be that the deposited gold film was too thick to be immobilized onto the electrode surface firmly enough and fell off during the repeated testing. Therefore, 300 s was chosen as the enrichment time.

### 3.3. Calibration Comparison Test of Ultramicro Electrode and Columnar Electrode

#### 3.3.1. Calibration of the Ultramicro Electrode by SWV

**Figure 8:**
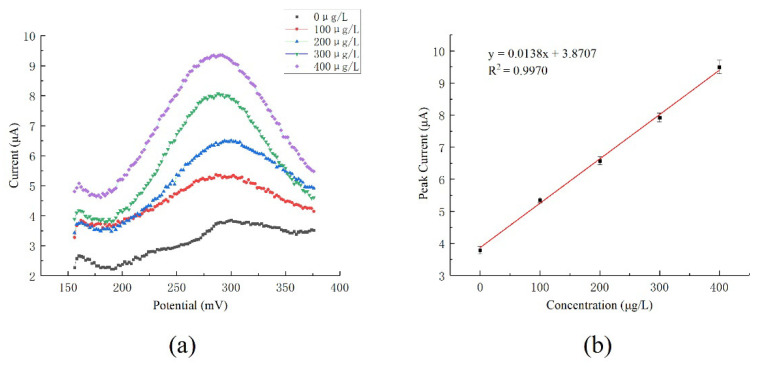
Cu^2+^ concentration detection by the ultramicro electrode: (**a**) Cu^2+^ concentration response curve; (**b**) Cu^2+^ current–concentration fitting line. (The detection results are the average of three times.).

The concentration of copper ions in water was detected by the developed system. Acetic acid and sodium acetate were used to prepare an acetic acid-sodium acetate buffer solution with pH = 4.5. A buffer and a copper standard solution with a standard value of 1000 μg/mL were used to prepare copper ion samples with copper ion concentrations of 100 μg/L, 200 μg/L, 300 μg/L, and 400 μg/L. The ultramicro interdigital electrode modified with gold nanoparticles was used to detect copper samples. According to the optimized experiment, the enrichment voltage of −0.5 V and the enrichment time of 300 s were chosen for detection. The specific detection process is as follows. Firstly, a voltage of 0.6 V was applied for 100 s to clean the electrode surface, in order to remove the residual copper. Then, a voltage of −0.5 V was applied for 300 s for enrichment. After that, the solution was left to stand for 3 s to reach an equilibrium state. Then, the dissolution of copper was carried out to obtain the dissolution curve. A detection process took approximately 7 min with the sample volume of 10 mL. The detection circuit system was used to apply the potential, to record the current responses, and to analyze the results. The current–concentration fitting line for copper ions determination is shown in Figure 8, which shows good linearity.

The linear response range to the copper ion standard solution was from 0 μg/L to 400 μg/L. According to the test results, the detection sensitivity of the ultramicro electrode was 0.0138 μA·L·μg^−^^1^, and the linear correlation coefficient was 0.9970. According to 3σ, the lower limit of detection was calculated as 18.89 μg/L.

#### 3.3.2. Comparison of Performance with Columnar Electrode

For comparison, a columnar glassy carbon electrode modified with gold nanoparticles, and a columnar platinum electrode were used as the working electrode and the counter electrode, respectively, to detect copper ion solutions with concentrations of 0 µg/L, 100 µg/L, 200 µg/L, 300 µg/L, and 400 µg/L. The developed circuit system was also used to complete the measurement.

**Figure 9:**
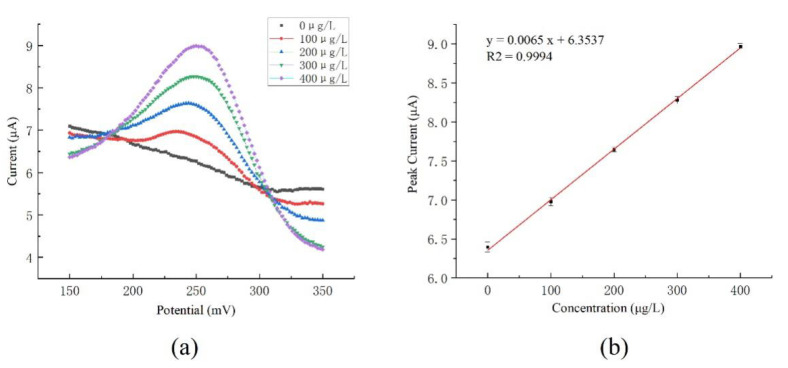
Cu^2+^ concentration detection by columnar electrode: (**a**) Cu^2+^ concentration response curve; (**b**) Cu^2+^ current–concentration fitting line (The detection results are the average of three times).

According to the testing curve, the Cu^2+^ current–concentration fitting line was shown in Figure 9. The peak value was obtained near the potential of 250 mV, and the peak currents with concentration values were fitted linearly. The linear response range of the columnar electrode to the copper ion standard solution was from 0 μg/L to 400 μg/L. According to the test results, the detection sensitivity of the columnar electrode was 0.0065 μA·L·μg^−1^, with the linear correlation coefficient of 0.9994. According to 3σ, the lower limit of detection was calculated as 29.76 μg/L.

According to the comparison experiment between the columnar glassy carbon electrode and ultramicro electrode, the detection sensitivity per unit area of ultramicro electrode was about 33.4 times higher than that of the columnar electrode, and it also showed a lower detection limit. The comparison results are shown in Table 2.

### 3.4. Anti-Interference Test

**Figure 10:**
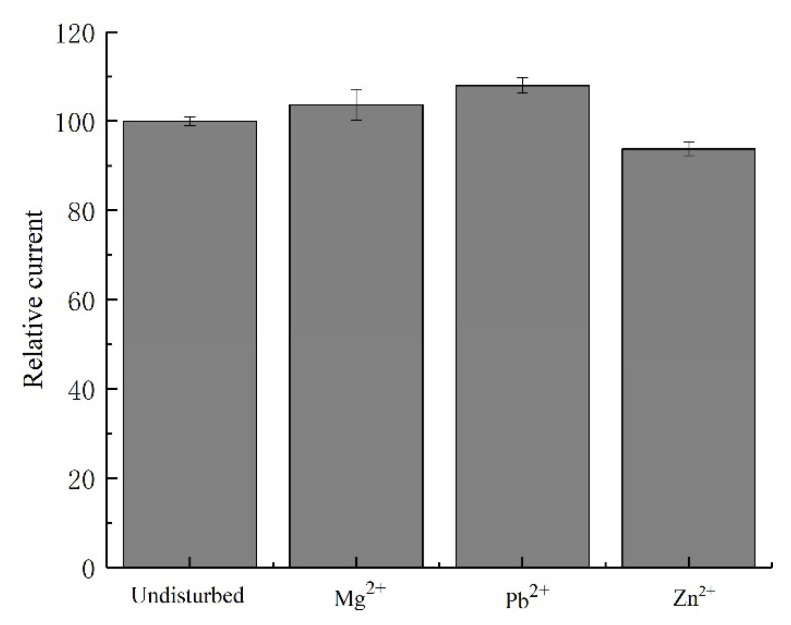
Influence of different interfering ions on response current.

When detecting copper ions Cu^2+^ in water, coexisting metal ions will interfere with the detection results. The anti-interference ability of the sensor electrode chip was tested by lead ions (Pb^2+^), magnesium ions (Mg^2+^), and zinc ions (Zn^2+^). Magnesium ions, lead ions, and zinc ions, with concentrations of 2 mg/L separately, were added into a 200 μg/L copper ion solution, and the corresponding current responses of the sensor electrode chip were measured. The response currents were compared with that of the 200 μg/L copper ion solution without interfering ions, and the test results are shown in Figure 10.

The deviations of current response caused by adding these three kinds of interference ions were within 10%, which indicated that the sensor electrode chip has an acceptable anti-interference ability with respect to magnesium ions, lead ions, and zinc ions.

### 3.5. Detection of Copper Ions in Real Water Sample

The real water samples taken from a municipal lake in Beijing were sent to the Pony Testing Company, and the concentrations of copper ions in these samples were detected by ion chromatography. The detection result showed that the concentration of copper ions in the lake was lower than the detection limit of 40 μg/L of the standard method, and the concentration of copper ions in the water sample could not be detected by ion chromatography. Therefore, the standard addition method was used for further detection. During the standard addition experiment, we considered that the concentration of the copper ion was zero. The real water sample was used to prepare an acetic acid–sodium acetate buffer solution with a pH value of 4.5, and then the buffer solution was used to prepare calibration solutions with different copper concentrations. The developed system was used for detecting the copper concentrations of the prepared sample. The straight-line equation of the calibration curve obtained by the standard addition was y = 0.017x + 4.878. Using the calibration curve, the spiked solutions with concentrations of 150 μg/L, 250 μg/L, and 350 μg/L were detected. The detection results of the standard addition experiment are shown in Table 3.

As shown in Table 3, the recoveries were between 87.5% and 94.7%. It was found that the portable heavy-metal-ion detection system developed in this study could be further used for the detection of heavy-metal ions in water.

## 4. Conclusions

In this study, a portable detection system with a nano-gold-modified, ultramicro interdigital electrode chip was designed and developed for rapid on-site detection of heavy-metal ions in water. The ultramicro electrode showed higher detection sensitivity and lower detection limit than of the columnar electrode. It exhibited a linear response to copper ions with the concentration range from 0 to 400 μg/L, and a good anti-interference ability with respect to other heavy-metal ions. A real water sample was detected using the standard addition method with the developed system; test results verified that the system had a potential ability for heavy-metal-ion detection. Although ultrasensitive detection was not achieved in this study, further work will focus on improving the performance of the sensor and the accuracy of the detection circuit.

## Figures and Tables

**Table 1:** WHO standards on the concentration of heavy-metal ions in drinking water.

Index	Limit Value (mg/L)	Index	Limit Value (mg/L)
Barium (Ba)	0.7	Lead (Pb)	0.01
Copper (Cu)	2	Manganese (Mn)	0.5
Hydrargyrum (Hg)	0.001	Zinc (Zn)	3

**Table 2:** Comparison of the detection sensitivity of different kinds of electrodes.

The Kind of Electrode	Effective Area of Electrode (mm^2^)	Sensitivity (μA·L·μg^−^^1^)	Sensitivity per Unit Area (μA·L·μg^−^^1^·mm^−^^2^)	Low Limit of Detection (μg/L)	Detection Range (μg/L)
columnar electrode	7.065	0.0065	0.0009	29.76	0–400
ultramicro electrode	0.450	0.0138	0.0307	18.89	0–400

**Table 3:** Adding standard recovery test results.

Sample	Added (μg/L)	Detection Results of This Study * (μg/L)	Recovery (%)
Lake water	150	138.53 ± 12.96	92.3%
	250	218.73 ± 4.90	87.5%
	350	331.47 ± 12.68	94.7%

* The detection results are the average of three times.
